# Supplementary material for: A UMLS-based spell checker for natural language processing in vaccine safety
Source: BMC Med Inform Decis Mak. 2007 Feb 12;7:3. doi: 10.1186/1472-6947-7-3 (PMC1805499; doi:10.1186/1472-6947-7-3)
Supplement: Additional file 13 — RAP application directory. Sets up directory for RAP (RDF application for PHP) source code [file 1472-6947-7-3-S13.gz › rap/test/test_TraverseModel.php]

Test Traverse MemModel

php
define("RDFAPI\_INCLUDE\_DIR", "./../api/");
include(RDFAPI\_INCLUDE\_DIR . "RdfAPI.php");
// Filename of an RDF document
$base="example1.rdf";
// Create a new MemModel
$model = new MemModel();
// Load and parse document
$model-load($base);
// Get Iterator from model
$it = $model->getStatementIterator();
// Traverse model and output statements
while ($it->hasNext()) {
$statement = $it->next();
echo "Statement number: " . $it->getCurrentPosition() . "  
";
echo "Subject: " . $statement->getLabelSubject() . "  
";
echo "Predicate: " . $statement->getLabelPredicate() . "  
";
echo "Object: " . $statement->getLabelObject() . "

";
}
// Move to the last statement and print it
$it->moveLast();
$statement = $it->current();
// Traverse model backward and print statements
echo $statement->toString() . "  
";
while($it->hasPrevious()) {
$statement = $it->previous();
echo $statement->toString() . "  
";
}
// Jump to statement 2 and print it
$it->moveTo(2);
$statement = $it->current();
echo $statement->toString() . "  
";
?>
